# Short-term outcomes following mini-open repair of chronic gluteus medius tendon tears using a double-row technique

**DOI:** 10.1093/jhps/hnab060

**Published:** 2021-08-25

**Authors:** Marc Barrera Uso, Hugo Bothorel, Lazaros Poultsides, Panayiotis Christofilopoulos

**Affiliations:** Orthopedic Department, La Tour Hospital, Avenue J.-D. Maillard 3, Meyrin, Geneva CH-1217, Switzerland; Research Department, La Tour Hospital, Avenue J.-D. Maillard 3, Meyrin, Geneva CH-1217, Switzerland; Academic Orthopaedic Department, Aristotle University Medical School, General Hospital Papageorgiou, Agiou Pavlou 76, Pavlos Melas, Thessaloniki GR-56429, Greece; Centre of Orthopaedic and Regenerative Medicine (CORE), Center for Interdisciplinary Research and Innovation (CIRI)-Aristotle University of Thessaloniki (AUTH), Balkan center, 10th km Thessaloniki-Thermi Rd, Thessaloniki GR-57001, Greece; Orthopedic Department, La Tour Hospital, Avenue J.-D. Maillard 3, Meyrin, Geneva CH-1217, Switzerland

## Abstract

Gluteal tendon tears represent a common but underreported cause of lateral hip pain and dysfunction. In case of conservative management failure, a surgical procedure must be performed to relieve patient symptoms. Current operative treatments, either open or endoscopic, have been however associated with different drawbacks which led to the introduction of the mini-open technique. The aim of this study was to evaluate and report the short-term outcomes of patients operated through the aforementioned surgical technique for gluteus medius (GM) chronic tears. We retrospectively analysed the records of 14 consecutive patients operated at the La Tour hospital by mini-open repair using a double-row technique for full-thickness GM chronic tears. Intra- and post-operative complications were recorded. The pre- and post-operative pain on visual analogue scale (pVAS), modified Harris Hip score (mHHS), abduction strength and gait dysfunction were assessed for all patients. Pre- and post-operative values were compared to evaluate whether improvements were statistically significant and clinically relevant. The study cohort comprised 13 women (93%) and 1 man (3%) aged 62.4 ± 18.0 at index surgery. No intra- or post-operative complications were noted. Compared to pre-operative values, patients reported a significant improvement in mHHS (59.1 ± 7.1 vs 92.7 ± 4.6) and pVAS (7.4 ± 1.0 vs 1.3 ± 1.3) at last follow-up. Patients exhibited a perfect improvement in muscle strength (3.6 ± 0.5 vs 5.0 ± 0.0), and the proportion of patients with a positive Trendelenburg sign decreased from 71% to 0%. Mini-open repair of chronic GM tendon tears using a double-row technique demonstrated excellent clinical and functional outcomes at short follow-up.

**Level of Evidence** IV.

## INTRODUCTION

Lateral hip pain localized at the greater trochanter has often be reported as a clinical manifestation of trochanteric bursitis. However, recent studies revealed that such a condition, also called greater trochanteric pain syndrome (GTPS), could rather be caused by gluteal tendinopathy or external snapping hip in more than half of the cases [1–3]. Tendon tears of the hip abductors [e.g. gluteus medius (GM) and gluteus minimus] constitute a common but underreported source of peritrochanteric pain, irritation, muscle weakness and gait dysfunction [4–7]. They occur in a considerable proportion of the middle-aged population, notably in women for whom the incidence reaches 25%, compared to only 10% in men [8, 9]. A recent literature review furthermore underlined that abductor tendinopathy increases with age, with a prevalence exceeding 80% in elderly patients (≥70 years) without hip-related problems [10], thus emphasizing the importance of systematic and thorough patient examination for avoiding misdiagnosis [11].

Initial GTPS treatment consists of nonsteroidal anti-inflammatory drugs (NSAIDs), physical therapy for strengthening and stretching of the hip abductors and, if necessary, corticosteroid injections into the greater trochanteric (GT) bursa. In case of conservative management failure, a surgical procedure may be performed to relieve patient symptoms [12]. Different techniques have been proposed, including endoscopic or open surgeries, both providing good to excellent clinical outcomes [11]. Endoscopic repair has been, however, associated with the difficult assessment of under-surface tears due to insufficient visibility and inability to correctly place anchors or mobilize the muscle-tendon complex [13]. On the other side, open repair has been associated with greater rates of complications (re-tears, infections, deep vein thrombosis and hematomas) and is, moreover, less cosmetic due to larger incisions [14–16].

Recently, the mini-open repair using a double-row (Speed Bridge) technique has been described and used to avoid the aforementioned limitations while granting satisfactory outcomes [13]. More published data on its clinical and functional results would be needed to evaluate whether mini-open repair is safe and efficient. The aim of our study was, therefore, to evaluate and report the short-term outcomes of patients operated through the aforementioned surgical technique for GM chronic tears.

## MATERIALS AND METHODS

### Patients

The authors retrospectively analysed the records of 31 consecutive patients operated for full-thickness GM chronic tears. All patients were operated by the same senior surgeon (PC) at the La Tour hospital (Geneva, Switzerland) between May 2018 and May 2020. The inclusion criteria were (i) the presence of a lateral thigh and chronic abductor insufficiency confirmed clinically and by magnetic resonance imaging (MRI) and (ii) the failure of previous non-operative treatments. Patients were, however, excluded if they had (i) symptomatic osteoarthrosis or (ii) previous surgeries on the symptomatic hip, (iii) a fatty GM degeneration of above stage 2 according to the Goutallier classification [17], (iv) other pathologies that could influence the study outcomes and (v) if they refused to participate in this study. All patients gave their written informed consent and the study protocol was approved by the ethics committee of Geneva (#2020-01885).

### Pre-operative evaluation

Trendelenburg gait and the level of muscle strength were evaluated for all patients. Abduction strength was evaluated with the patient lying on the contralateral side and the affected side abducted against resistance and was graded using the ordinal Medical Research Council (MRC) scale (0, No contraction; 1, Flicker or trace of contraction; 2, Full range of active movement, with gravity eliminated; 3, Active movement against gravity; 4, Active movement against gravity and resistance; 5, Normal power). The modified Harris Hip Score (mHHS) from 0 (worst) to 100 (best) and pain on visual analogue scale (pVAS) from 0 (best) to 10 (worst) were also recorded.

### Surgical technique

**Fig. 1.**
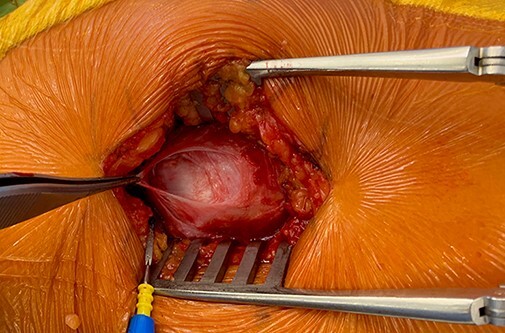
After incising the fascia lata and excising the trochanteric bursa, we observe a full thickness tear of the gluteus medium tendon. The forceps is situated in the cephalic portion.

**Fig. 2.**
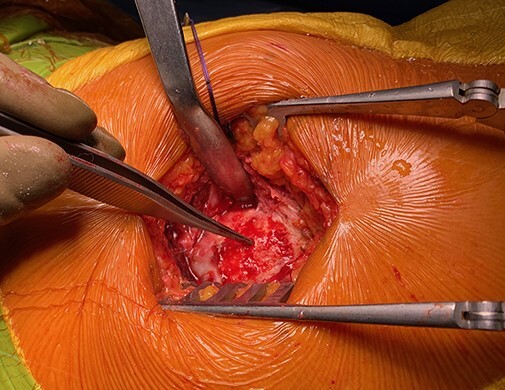
The degenerative tissue and osteophytes of the anterolateral facette of the GT are cleared and the tendon footprint is recreated using a round burr.

**Fig. 3.**
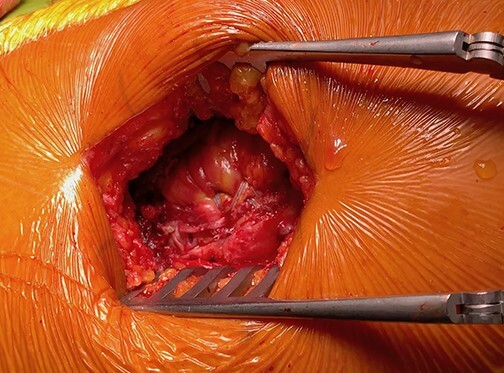
Double-row, crossed-limb reconstruction.

The patients were placed in the lateral decubitus position and maintained with positioners placed on the pubic symphysis and sacrum. A 5–10 cm incision was performed and centred over the greater trochanter following the anatomic axis of the femur. Following the dissection and retraction of the subcutaneous tissue, the fascia lata was incised longitudinally over the greater trochanter to reveal the tear location (Fig. 1). It is worth noting that tear identification can be challenging due to the presence of important scar tissue. For these cases, a solution comprising 20 cl of NaCl was injected over the theoretical insertion of the GM (blow test) for a better lesion identification on the sagittal MRI view. Release and mobilization were then performed and the free end of the GM was whipstitched with an Ethibond 3 (also for the gluteus minimus if possible). The greater trochanter area was then exposed and the footprint identified (Fig. 2). The GT was prepared using a round burr over an area of 2–3 cm^2^; the preparation aimed to reveal cancellous bone to facilitate muscle flaps healing. To not disturb the vascular supply of the femoral head, three drill holes (2.0 mm diameter) were made at the anterior and posterior margins of the footprint as close as possible to the normal insertion. Then, four 4.75 mm SwiveLock C anchors (Arthrex, Naples, FL, USA) were double loaded and inserted (Fig. 3). An Arthrex FiberTape (Arthrex, Naples, FL, USA) was used. The sutures were then passed through the GM and gluteus minimus, transferring their insertion on the major trochanter with the hip in 15°–20° of abduction. Apart from abduction, internal rotation was also needed to counteract natural anteversion. The sutures were tied such that no gaps could remain between the anchors and the tendon.

### Post-operative rehabilitation

The patients were educated to walk with a partial weight-bearing of 15 kg using two crutches for the first eight post-operative weeks with no active abduction and passive adduction. Physiotherapy was initiated at 2 months post-operatively, performing active abduction against gravity, gait training and progression to full weight-bearing. The patients also received a standard anticoagulation therapy during the first four post-operatives weeks.

### Intra- and post-operative evaluation

All intra- and post-operative complications related to the surgery were documented. All patients were evaluated post-operatively at 6 months and 1 year at our hospital to evaluate the pVAS, mHHS, muscle strength and Trendelenburg gait. Patients were contacted by mail or telephone to update their records (only for pVAS and mHHS).

### Sample size calculation and statistical analyses

Maldonado *et al*. reported a pre-operative mHHS of 54.7 ± 15.9 points for patients with full thickness GM tears [18]. The minimal clinically important difference (MCID) of the mHHS at short follow-up was reported to be 13 points [19]. *A priori* sample size calculation to ensure the fulfilment of the principal goal of the study indicated that 14 patients would be needed to detect a difference in mHHS of 13 points with a standard deviation of 15.9 points and a statistical power of 0.80.

For baseline characteristics, variables were reported as mean ± standard deviation or proportions. Shapiro–Wilk tests were used to assess the normality of distributions. Differences between pre-operative and post-operative values (mHHS, pVAS and Hip strength) were evaluated using either the paired student *t*-test (if Gaussian distribution) or the Wilcoxon signed-rank test (if non-Gaussian distribution). For subgroup analyses (mHHS, pVAS and hip strength improvements), differences between groups were evaluated using Wilcoxon rank-sum tests (Mann–Whitney *U*-test) or unpaired student *t*-test. The improvement in mHHS was compared to the MCID set at 13 points, while the post-operative mHHS was compared to the patient acceptable symptom state (PASS) set at 77.5 points [20]. For categorical data, differences between groups were evaluated using the Fisher exact test. Statistical analyses were performed using R version 3.6.2 (R Foundation for Statistical Computing, Vienna, Austria). *P*-values <0.05 were considered statistically significant.

## RESULTS

From the initial cohort of 31 patients, 9 (29%) were excluded because of previous hip surgeries, 6 (19%) because of a fatty GM degeneration of above stage 2, 1 (3%) because of a spinal pathology that needed local lumbar infiltrations and 1 (3%) refused to participate in this study. None of the patients were lost to follow-up. This left a final cohort of 14 patients with up-to-date records at a follow-up of 10.6 ± 4.8 months (median, 10.0; range, 6.0–18.0) for their last clinical visit and 14.3 ± 7.0 months (median, 14.5; range, 6.0–24.0) for their last updated PROMs. The study cohort comprised 13 women (93%) and 1 man (3%) aged 62.4 ± 18.0 (range, 18–86) at index surgery. Seven patients (50%) underwent both GM and gluteus minimus reattachments, while 7 patients (50%) had isolated GM repair only due to the absence of gluteus minimus lesions (*n* = 2) or excessive fatty gluteus minimus degeneration (n = 5). Other patient pre-operative characteristics, including symptoms onset before surgery, presence of gluteus minimus lesions or radiologic osteoarthrosis, as well as fatty GM degeneration stage, are presented in Table I.

**Table I.** Patient characteristics

	*Total (n = 14 patients)*	
	*Mean ± SD*	
	*n (%)*	*Median (min–max)*
Age (years)	62.4* *±* *18.0	66.5 (18.0*–*86.0)
Symptoms onset^a^ (months)	16.6* *±* *14.0	12.0 (2.0*–*36.0)
Gender		
Men	1 (7%)	
Women	13 (93%)	
Gluteus minimus lesions	12 (86%)	
Gluteus minimus reattachment	7 (50%)	
Osteoarthrosis		
None	10 (67%)	
Tonis I	4 (27%)	
Fatty GM degeneration		
Stage 0	5 (40%)	
Stage 1	6 (40%)	
Stage 2	3 (20%)	

a Preceding surgery; GM, gluteus medius.

### Patient outcomes

None of the patients presented wound complications or experienced other intra- or post-operative complications.

Compared to pre-operative values, patients reported a significant improvement in mHHS (59.1 ± 7.1 vs 92.7 ± 4.6; *P* < 0.001) and pVAS (7.4 ± 1.0 vs 1.3 ± 1.3; *P* < 0.001) at last follow-up (Table II). All patients improved their mHHS beyond the MCID (>13 points) with a post-operative mHHS exceeding the PASS (>77.5 points). Patients exhibited a considerable improvement in abduction strength (3.6 ± 0.5 vs 5.0 ± 0.0; *P* < 0.001), and the proportion of patients with a positive Trendelenburg sign decreased from 71% (*n* = 10) pre-operatively to 0% at their last follow-up visit. All patients improved their abduction strength by at least one grade. It is worth noting that patients who benefited from adjuvant gluteus minimus reattachment did not differ from those with isolated GM repair in terms of mHHS, pVAS or strength improvements (*P* = 0.439, *P* = 0.340 and *P* = 0.293, respectively).

**Table II.** Pre- and post-operative outcomes

	*Total (n = 14 patients)*	
	*Mean ± SD*	
	*n (%)*	*Median (min*–*max)*
Modified Harris Hip Score (mHHS)		
Pre-operative	59.1* *±* *7.1	58.5 (47.0–72.0)
Post-operative	92.7* *±* *4.6	93.5 (85.0–98.0)
Improvement	33.6* *±* *6.5	34.5 (20.0–49.0)
*P*-value	0.001	
Pain on visual analogue scale (pVAS)		
Pre-operative	7.4 ± 1.0	8.0 (6.0–9.0)
Post-operative	1.3 ± 1.3	1.0 (0.0–4.0)
Improvement^a^	6.1 ± 0.9	6.0 (4.0–7.0)
*P*-value	<0.001	
Abduction strength		
Pre-operative	3.6 ± 0.5	3.8 (3.0–4.0)
Post-operative	5.0 ± 0.0	5.0 (5.0–5.0)
Improvement	1.4 ± 0.5	1.3 (1.0–2.0)
*P*-value	<0.001	
Trendelenburg sign		
Pre-operative	10 (71%)	
Post-operative	0 (0%)	
Improvement	100%	
*P*-value	<0.001	

a A positive improvement indicates a decrease in pVAS.

## DISCUSSION

The most important finding of this study was that mini-open repair using a double-row technique demonstrated excellent clinical and functional outcomes at short follow-up for patients suffering from chronic full-thickness GM tears with low fatty muscle degeneration. In comparison to single-row repair, the double-row technique is known to grant stronger biomechanical properties, better tendon healing and lower retear incidence as underlined by several studies on rotator cuff tears [21–24]. Moreover, conversely to endoscopic surgeries, the mini-open repair offers a better tear visualization, a reconstruction that is closer to patients’ anatomy, and provides the flexibility to perform another procedure than the one planned in case of intraoperative assessment of irreparable tears.

Gluteal tendon tears represent a common but underreported cause of hip pain and dysfunction [25]. While such lesions have been reported following total hip arthroplasty [8, 26–28] or traumatic events [5, 29, 30], the prevalence of chronic tears in patients suffering from GTPS is high and should not be ignored. To date, these degenerative and inflammatory lesions become a matter of particular interest [31–36], notably, because of the global population ageing related to advances in medical technologies. Different authors, therefore, emphasized the importance of an early and adequate diagnosis using either imaging results or clinical tests to facilitate orthopaedic care [7, 37]. Current surgical treatments, either open or endoscopic, have been, however, associated with different limits and drawbacks which led to the introduction of the mini-open technique [13]. In the present study, the authors analysed a consecutive series of patients operated for chronic GM tears and demonstrated the efficacy of mini-open repair at alleviating patients’ symptoms at short follow-up.

The short-term clinical outcomes of the present series were excellent. The improvement in mHHS (34 points) was comparable to those reported in the recent literature at a minimum of 2 years of follow-up for open or endoscopic repair of gluteal tendon tears (18.4–46.5 points; Table III) [13, 18, 20, 38–43]. All patients included in the present series (100%) met the PASS in terms of mHHS, which compares favourably with the results of Maldonado *et al.* [18] (69%) and Kirby *et al.* [40] (88%). To the authors’ knowledge, the post-operative mHHS reported in our series (93 points) is above every single result published in the existing literature (68–86 points) at different follow-up time points [13, 14, 17, 18, 20, 38–43]. Likewise, the post-operative pain reported in the present series (median, 1) is one of the greatest (lowest) values reported in the literature, which ranges from 0.5 to 3.4 points [13, 14, 17, 18, 20, 38, 39, 41–43]. This is of particular interest because such a low post-operative pain level is known to be associated with a considerable reduction of opioid consumption [44]. Improvements in PROMs seem to be associated with functional results given that all our patients (100%) improved their abduction strength by at least one grade, which is greater than the proportions reported by Maldonado *et al.* [18] (44%), Nazal *et al.* [41] (53%) and Hartigan *et al.* [39] (64%). The present series also revealed the complete disappearance of gait dysfunction at last clinical evaluation, which is consistent with the findings of Hartigan *et al.* [39] who observed Trendelenburg gait disappearance in 86% of his patients.

**Table III.** Literature review of recent studies evaluating outcomes following surgical repair of gluteal tendon tears

*Part A*									
							*Tears*		
*Study*	*Year*	*Journal*	*N*	*Women*	*Mean* *Age (yrs)*	*Surgical procedure*	*Degenerative etiology*	*Tendons*	*Type*
This study	2021	*J Hip Preserv Surg.*	14	93%	63	Mini-open	100%	GM ± Gm	FT
DeFroda *et al*.	2019	*J Hip Preserv Surg.*	31	87%	59	Mini-open	–	GM ± Gm	FT + P
Maslaris *et al*.	2020	*Journal of Arthroplasty*	23	87%	68	Open	83%	GM ± Gm	FT + P
Maldonado *et al*.	2020	*Orthop J Sports Med.*	36	86%	65	Open	–	–	FT
Maslaris *et al*.	2020	*Journal of Arthroplasty*	10	70%	59	Endoscopic	80%	GM ± Gm	FT + P
Nazal *et al*.	2020	*Arthroscopy*	15	80%	67	Endoscopic	93%	GM ± Gm	FT
Kirby *et al*.	2020	*Arthroscopy*	20	79%	51	Endoscopic	100%	–	FT + P
Okoroha *et al*.	2019	*Am J Sports Med.*	60	92%	58	Endoscopic	–	GM ± Gm	P
Hartigan *et al*.	2018	*Arthroscopy*	25	96%	54	Endoscopic	–	GM	P
Thaunat *et al*.	2018	*Arthroscopy*	20	85%	66	Endoscopic	–	GM	FT + P
Perets *et al*.	2017	*Arthroscopy*	16	93%	57	Endoscopic	–	GM	FT + P
Bogunovic *et al*.	2015	*Arthroscopy*	30	90%	62	Endoscopic	–	GM ± Gm	FT + P
Chandrasekaran *et al*.	2015	*J Bone Joint Surg Am.*	34	94%	57	Endoscopic	100%	GM	FT + P
Dominguez *et al*.	2015	*Arch Orthop Trauma S.*	23	83%	51	Endoscopic	–	GM	–
*Part B*									
					*Mean mHHS*		*Mean pVAS*		
*Study*	*Fatty GM degeneration*	*Mean FU (months)*			*Post-op*	*Improv.*	*Post-op*	*Improv.*	
This study	Stage ≤2	14			93	34	1.3	6.1	
DeFroda *et al*.	–	6			68^a^	22^a^	3.5^a^	2.8^a^	
Maslaris *et al*.	All stages	22			–	–	–	4.1	
Maldonado *et al*.	–	41			73	18	2.7	2.3	
Maslaris *et al*.	All stages	16			–	–	–	4.1	
Nazal *et al*.	All stages	31			83	29	2.4	3.0	
Kirby *et al*.	Stage ≤2	29			76	38	–	–	
Okoroha *et al*.	–	24			75	28	2.7	4.0	
Hartigan *et al*.	–	33			76	21	2.7	5.0	
Thaunat *et al*.	All stages	32			80	47	3.2	4.0	
Perets *et al*.	–	35			81	29	2.6	3.6	
Bogunovic *et al*.	All stages	35			81	–	1.7	–	
Chandrasekaran *et al*.	–	27			82	–	2.4	4.2	
Dominguez *et al*.	–	12			86	46	0.5	7.6	

GM, gluteus medius; Gm, gluteus minimus; FT, full thickness; P, partial; Post-op, post-operative; FU, follow-up; Improv., pre- to post-op improvement;

a Rescaled for adequate comparison.

Although such comparisons with the literature illustrate the satisfactory outcomes of mini-open repair, they remain, however, debatable because studies differ in terms of patients’ aetiology (degenerative, post-traumatic or post-operative tears), tear types (partial or full thickness tears), fatty muscle degeneration, surgical procedure (endoscopic, open or mini-open) or fixation technique (single or double row). Such a heterogeneity prevents us from identifying the best treatment option according to patient characteristics and tear patterns as recently underlined in a review article that aimed to develop clinical guidelines for open surgery of acute and chronic tears of hip abductor tendons [12]. The only study that described patient outcomes following a similar surgical technique is the one published by DeFroda *et al.* [13] who reported promising 6 months outcomes and potential benefits. However, such results are hardly interpretable because the aforementioned pilot study lacks information and clarity on patients and tears characteristics. Our study is, therefore, the first to investigate the clinical and functional benefits of mini-open repair using a double-row technique on a well-described and homogeneous population (chronic full-thickness GM tears with low fatty muscle degeneration). It is worth noting that the cohort size of the present study, which might seem low (14 patients), is relatively high compared to other published series, notably when considering our strict inclusion and exclusion criteria [11]. Only patients who had acute symptoms, a good muscle quality and minor retraction on MRI were selected from our initial cohort of 31 cases, which probably explains our good results compared to others. The excellent results presented in this study, therefore, reflect both the efficiency of the surgical technique and an adequate patient selection.

Tendon repair using a double-row technique has been largely studied for rotator cuff tears in the shoulder joint. Different authors reported that such a procedure could lead to a specific type of retear (Type 2—medial cuff failure) potentially induced by the medial transfer of the tension-bearing row and the oblique passage of instruments through the tendon, which creates larger holes and more tension on the medial side [45–47]. Furthermore, Christoforetti *et al.* [48] reported that a second row of suture anchors could reduce the intratendinous blood flow by nearly 50% at the time of initial fixation. Further studies with a longer follow-up and greater cohort size are, therefore, needed to evaluate the optimal surgical technique in terms of biomechanical and biological properties.

This study presents, however, several limitations. First, its retrospective nature and absence of a comparative or control group. Second, the functional outcomes (abduction strength and gait dysfunction) and PROMs (mHHS and pVAS) were not assessed at a similar follow-up time point since only the latter could be updated by mail or telephone. Additional clinical visits at greater follow-up are, therefore, needed to evaluate whether patient improvements in abduction strength and gait dysfunction are stable over time. Furthermore, the last follow-up was not consistent across patients, ranging from 6 to 24 months. While the authors usually evaluate patients at the hospital, this remote PROMs evaluation had the advantage to reduce costs, patient travels and potential risks related to the coronavirus disease-19 (COVID-19). For similar reasons, the authors did not perform post-operative MRI examination of tendons’ repair integrity. Lastly, the authors used the mHHS to clinically follow patients with gluteal tendon tears, while this PROM was initially designed for osteoarthritis. The mHHS remain, however, commonly assessed in routine for many hip pathologies and is often useful for comparisons with existing published studies.

## CONCLUSION

Mini-open repair of chronic GM tendon tears using a double-row technique demonstrated excellent clinical and functional outcomes at short follow-up. This procedure seems to be an adequate compromise between endoscopic and traditional open surgeries since it grants an appropriate tear visualization and a small incision.

## DATA AVAIILABILITY

The data underlying this article cannot be shared publicly for the privacy of individuals that participated in the study.
