# Correction to: Short-term outcomes following mini-open repair of chronic gluteus medius tendon tears using a double-row technique

**DOI:** 10.1093/jhps/hnae012

**Published:** 2024-02-28

This is a correction to: Marc Barrera Uso, Hugo Bothorel, Lazaros Poultsides, Panayiotis Christofilopoulos, Short-term outcomes following mini-open repair of chronic gluteus medius tendon tears using a double-row technique, *Journal of Hip Preservation Surgery*, Volume 8, Issue 2, July 2021, Pages 202–208, https://doi.org/10.1093/jhps/hnab060)

The following change has been made to the original publication of this manuscript. The first author’s name has been emended to read: ‘Marc Barrera Uso’ instead of: ‘Marc Barrera’. The emendation has been made in the article.

